# Enrichment of A Rare Subpopulation of miR-302-Expressing Glioma Cells by Serum Deprivation 

**DOI:** 10.22074/cellj.2015.495

**Published:** 2015-01-13

**Authors:** Mahmoud-Reza Rafiee, Afsaneh Malekzadeh Shafaroudi, Sara Rohban, Hamid Khayatzadeh, Hamid Reza Kalhor, Seyed Javad Mowla

**Affiliations:** 1 Nanomedicine and Tissue Engineering Center, Shahid Beheshti University of Medical Sciences, Tehran, Iran; 2 Department of Non-coding RNA Research, Pars Genome Company, Tehran, Iran; 3 Department of Molecular Genetics, Faculty of Biological Sciences, Tarbiat Modares University, Tehran, Iran; 4 Department of Chemistry, Sharif University, Tehran, Iran

**Keywords:** Gene Expression, microRNA, miR-302, Glioma, Cancer Stem Cell

## Abstract

**Objective:**

MiR-302-367 is a cluster of polycistronic microRNAs that are exclusively expressed in embryonic stem (ES) cells. The miR-302-367 promoter is functional during embryonic development but is turned off in later stages. Motivated by the cancer stem cell hypothesis, we explored the potential expression of miR-302 in brain tumor cell lines.

**Materials and Methods:**

In the present experimental study, we have tried to expand our knowledge on the expression pattern and functionality of miR302 cluster by quantifying its expression in a series of glioma (A-172, 1321N1, U87MG) and medulloblastoma (DAOY) cell lines. To further assess the functionality of miR-302 in these cell lines, we cloned its promoter core region upstream of the enhanced green fluorescent protein (EGFP) or luciferase encoding genes.

**Results:**

Our data demonstrated a very low expression of miR-302 in glioma cell lines, compared with that of embryonal carcinoma cell line NT2 being used as a positive control. The expression of miR-302 promoter-EGFP construct in the aforementioned cell lines demonstrated GFP expression in a rare subpopulation of the cells. Serum deprivation led to the generation of tumorospheres, enrichment of miR-302 positive cells and upregulation of a number of pluripotency genes.

**Conclusion:**

Taken together, our data suggest that miR-302 could potentially be used as a novel putative cancer stem cell marker to identify and target cancer stem cells within tumor tissues.

## Introduction

Based on the cancer stem cell (CSC) hypothesis, tumors arise from a unique subset of tumor cells, exhibiting properties similar to stem cells. Such a link between cancer and stem cells is supported by the observations that many altered pathways in cancer cells normally contribute to the stemness property of embryonic and adult stem cells (1). A CSC is defined as an undifferentiated cell with the ability to self-renew and capability to partially differentiate to form all subpopulations within a tumor. This new concept could change our understanding of tumor development and progression, which may ultimately improve the current diagnostic and therapeutic approaches by allowing us to better identify and target CSCs (2). The aim of many ongoing investigations is to develop novel cancer stem cell-directed treatments, which could reduce therapy resistance, relapse and the toxicity associated with the current non-selective agents.

MicroRNAs (miRNAs, miRs) are a unique class of non-coding RNAs involved in diverse physiological and developmental processes including proliferation, differentiation, and apoptosis (3). MiRNAs are initially transcribed as larger precursors, which are then excised to produce mature forms of 20-22 nucleotides length (3). While miRNA genes constitute only 1-2% of known eukaryotic genes, they are estimated to regulate the translation of more than 60% of proteincoding genes, through sequence-specific complementary binding to their target mRNAs (mainly 3´ UTR) (3, 4). The expression of miRNAs is cell- and tissuespecific and the exclusive expression of some miRNAs in embryonic stem cells (ESCs) is one such case (5, 6). Various deregulations of miRNAs have been linked to tumorigenesis, where some misexpressed miRNAs can function as either oncogenes or tumor suppressors (7-9).

Recent efforts to define ESC-specific miRNAs have led to the discovery of several miRNA clusters which are expressed in undifferentiated ESCs and are turned off upon the induction of differentiation. The cluster of hsa-miR-302-367 is located on chromosome 4 and consists of nine members (miR-302b*, miR-302b, miR-302c*, miR-302c, miR-302a*, miR-302a, miR-302d, miR-367*, and miR-367) co-transcribed in a poly-cistronic manner (10). MiR-302s are exclusively expressed at high levels in ESCs indicating their essentiality for maintenance of self-renewal and pluripotency of stem cells. The promoter of miR-302-367 is turned on by ESC-specific transcription factors OCT4, SOX2, Nanog, and Rex1 (10, 11). Therefore, the expression of miR-302s can be used as a unique marker to explore the stemness state of the cells.

Recently, miR-302 has been implicated in reprogramming (11) and tumorigenesis (12). Based on the new proposed role for CSCs in tumorigenesis, it is important to examine the expression and involvement of stem cell-specific genes in cancer cells. In the present study, we have examined the potential expression of ESC-specific microRNA, miR-302, in four different brain tumor cell lines. We further investigated whether this expression was confined to a specific subpopulation of cells with stem cell properties.

## Materials and Methods

### Construction of the miR-302 promoter-GFP/Luciferase vectors

In this experimental study, a ~1200bp genomic segment corresponding to the human miR-302-367 promoter was amplified from HEK cells, using the following primers containing the BglII and HindIII restriction sites (underlined letters) respectively.

Forward: 5´ATTTAGATCTCAAGAGTAACACATCTGG3´

Reverse: 5´TATTAAGCTTCCCAAAGATTCGTGTTC3´

The amplified product was cloned either in the pEGFP-N1 vector replacing the CMV promoter or in the pGL3 vector replacing the SV40 promoter.

### Lentiviral vectors construction and transduction

7TGC vector (13) was digested with ClaI and NheI to replace its promoter region with miR-302- 367 or Nanog promoters. A T75 flask of 293T cells were transfected with the lentiviral vector, psPAX2 and VSV-G with the ratio of 10 μg, 7.5 μg and 2.5 μg respectively. About 12 hours after the infection, the media were replaced with fresh low-Glucose Dulbecco’s Modified Eagle’s Medium (DMEM), supplemented with 10% fetal bovine serum (FBS). The media containing the cells were collected after 24 hours, filtered by a 0.45 μm syringe filter, and were then subjected to ultrafiltration by Millipore Amicon ultra-15 centrifugal filters, for 10 minutes at 4000 g, to reduce the volume to about 200 μl. The concentrated viruses were then added to the media of the glioma cell lines, supplemented with 4 μg/ml Polybrene (Sigma, St. Louis, MO, USA). One day after the infection, the media of the target cells was discarded, and the cells were washed out with PBS.

### Cell line cultures and treatments

The human embryonic carcinoma cell line NT2 (NTERA2, as a positive control (14)) and human bone marrow stromal cells (BMSC), as negative control (15), were obtained as generous gifts from Drs. Andrews and Soleimani respectively. The human glioma cell lines U87MG, DAOY, A-172 and 1321N1 were obtained from Pasteur Institute of Iran (16) and grown in DMEM supplemented with 10% FBS, 4 mM L-glutamine pyruvate and 1% penicillin/streptomycin at 37˚C in a 5% CO_2_ atmosphere. All cell lines were transfected with Fugene HD transfection reagent (Roche, Germany) according to the manufacturer’s instructions. The expression of green fluorescent protein (GFP) and luciferase, as indicators for promoter activity of mir-302s, were monitored by fluorescent microscopy or luminometry (17), 24-48 hours after transfection.

### Luciferase assay

Luciferase activity was measured by a luciferase reporter assay system (Promega, WI, USA). Briefly, the cells transfected with pGL3-pmiR were washed and lysed prior to luciferase assay (17). All experiments were performed in duplicate or triplicate.

### Real-time polymerase chain reaction (PCR)

Total RNA from cell lysates was extracted with Trizol reagent and further treated by RNase-free DNase (Takara, Japan). The U6 snRNA gene was used as an internal control. The locked nucleic acid (LNA) primers for U6 and miR-302s were manufactured by Exiqon (Denmark). Briefly, two micrograms of total RNA was used for RT reaction, using a cDNA Reverse Transcription Kit (MiRCURY LNA*TM* Universal RT microRNA), according to the manufacturer’s instructions. Realtime PCR was performed with SYBR green master mix, Universal RT (Exiqon, Denmark) and micro- RNA LNATM primer sets, and analyzed with an ABI 7500 real-time PCR system. To quantify the expression level of pluripotency genes, a carefully designed set of primers (Table 1) were employed. RNA extraction, RT, and real-time PCR were performed as previously described (14).

### Statistical analysis

Fold changes in the expression levels were calculated with the formula Log_10_RQ=Log_10_ 2^−Δ(ΔCT)^. A Log_10_RQ=0 corresponds to no expression change, while a Log_10_RQ=1 means 10 times elevation in expression level compared to the internal control (GAPDH for protein-coding genes and U6 for microRNAs). All reactions were performed in duplicate or triplicate. Group-wise comparison and statistical analysis of the relative expression results of real-time PCR were carried out by REST 2008 Relative Expression Software Tool 2008 (REST, V2.0.7, Corbette Research Pty. Ltd.). Excel 2007 and GraphPad Instat3 were used to plot the charts. Student t test and ANOVA were used to analyze the significance of differences among different groups.

**Table 1:** The sequences and the PCR products sizes of the primers used to amplify selected ES-specific transcription factors


Gene	Primer sequence	PCR product size (bp)

**Oct4A**	F: CGCAAGCCCTCATTTCAC	111
	R: CATCACCTCCACCACCTG	
**Oct4B**	F: GTCTTCTGCCTTTTAAAATCCA	159
	R: GGCTGAATACCTTCCCAAATA	
**Oct4B1**	F: GGGTTCTATTTGGTGGGTTCC	128
	R: TCCCTCTCCCTACTCCTCTTCA	
**Sox2**	F: GACTGAGAGAAAGAAGAGGAGAG	161
	R: GAGAGAGGCAAACTGGAATC	
**Nanog**	F: TGCCCATCCAGTCAATCTCA	444
	R: TCCAGAGACGGCAGCCAAG	
**GAPDH**	F: GTGAACCATGAGAAGTATGACAAC	123
	R: CATGAGTCCTTCCACGATACC	


PCR; Polymerase chain reaction and ES; Embryonic stem.

## Results

### Members of miR-302 cluster are expressed at very low levels in brain tumor cell lines

**Fig 1:**
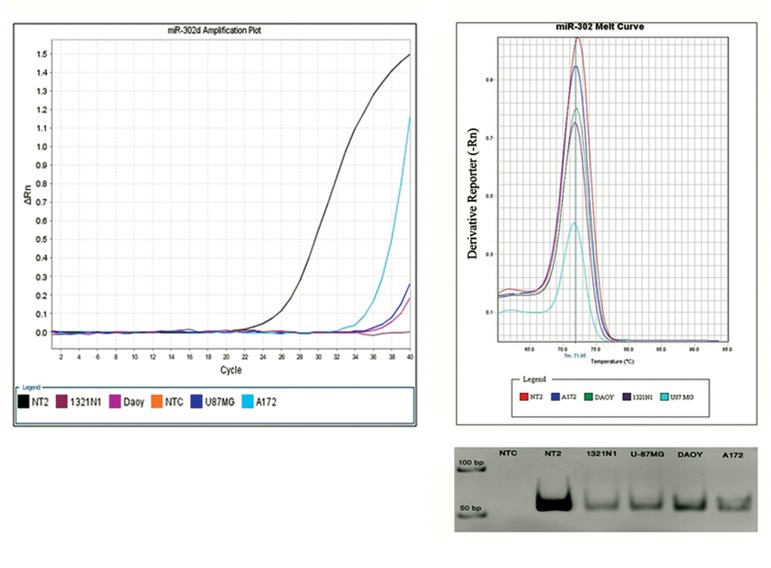
A. a SYBR Green based real-time amplification, using specific LNA primers for miR-302d, was used to quantify the expression of miR-302d in different glioma cell lines (1321N1, DAOY, U87MG and A172) as well as an embryonic carcinoma cell line NT2. Note that the expression level of miR-302d in glioblastoma cell lines is much lower than that of NT2. B. A typical plot of the dissociation curve for miR302d amplicons. Similar melting curves (tm=71.85˚C) of the miR-302b amplifications in NT2 and DAOY cell lines confirmed the authenticity of the PCR products. C. The obtained CT of miR-302 members in different glioma cell lines compared with that of the NT2 cell line. Note that the lower the CT, the higher the expression level. D. PCR products of miR-302b amplification in NT2, as a positive control, and glioma cell lines were electrophoresed on a 12% polyacrylamide gel. NTC lane represents the negative control lane.

The expression of miR-302 members (normalized to that of U6 snRNA) in brain tumor cell lines 1321N1, DAOY, A172 and U87MG was evaluated by means of real-time RT-PCR, employing commercially available LNA primer pairs for specific amplification of each member. The embryonal carcinoma cell line NT2 was used as a positive control to optimize the amplification of miR-302 members. As shown in figure 1A, B, the quantitative RT-PCR assay demonstrated a significantly lower level of miR-302s expression (p<0.001) in the glioma cell lines compared with their expression in NT2 cells (more than 18 CT difference). An identical and expected melting curve (Fig 1C) and product size (Fig 1D) of the PCR products in NT2 and the glioma cell lines, confirmed the authenticity of the amplified products. Among the cell lines, A172 showed the highest level of expression followed by DAOY, 1321N1 and U87MG respectively.

### MiR-302s are expressed in a rare subpopulation of glioma cell lines

**Fig 2:**
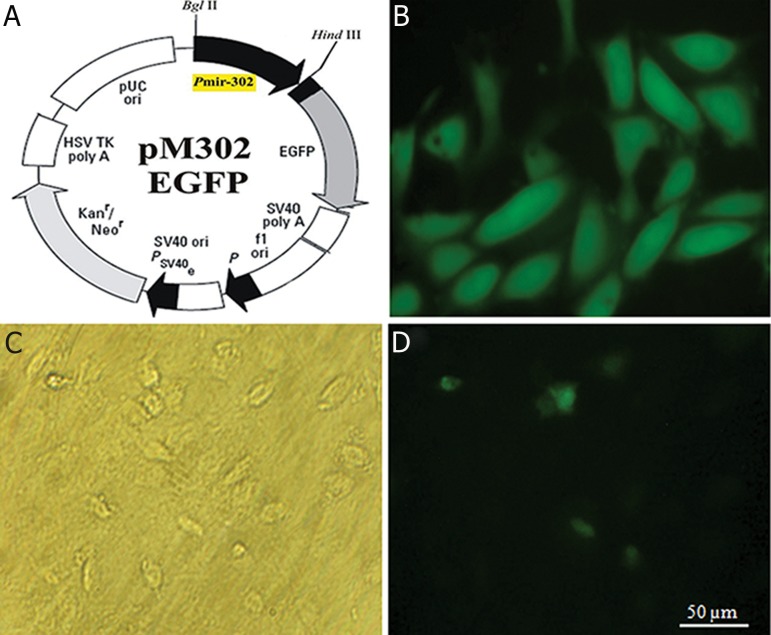
a pmiR-302-EGFP vector was employed to report the presence of miR-302 promoter activity in different cell lines. A. a ~1200 bp DNA fragment containing human miR-302 promoter was cloned into the pEGFP-N1 vector by means of standard molecular techniques. B. the NT2 cell line, as a positive control, was routinely transfected with the pmiR-302- EGFP vector, using Fugene HD transfection reagent. Presence of the GFP signal was observed in high percentage of the cells under a fluorescent microscope, 24-48 hours after transfection. C. A phase-contrast microscopy profile of the DAOY cell line, a human medulloblastoma cell line, was transfected with the pmiR-302-EGFP vector. D. Presence of GFP signals was observed in a few cells by fluorescent microscopy, 48 hours after transfection. EGFP; Enhanced green fluorescent protein.

Due to the very low expression of miR-302s in the glioblastoma cell lines, we constructed an expression vector in which the open-reading frame of the GFP had been cloned under the control of miR-302 promoter (Fig 2A). While most of the transfected NT2 cells were GFP-positive (Fig 2B), only a few transfected medulloblastoma cells (DAOY, Fig 2C) were GFP-positive (compare Fig 2D, C).

**Fig 3:**
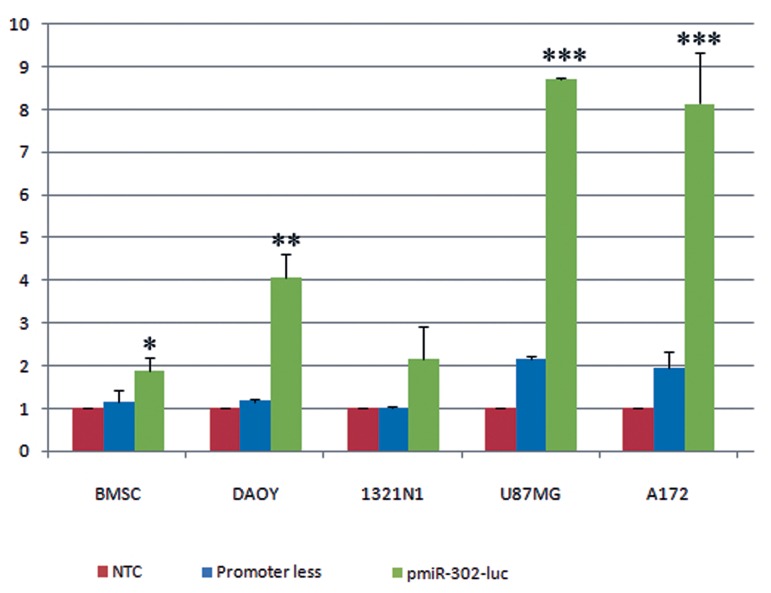
Transfected glioma cell lines as well as BMSCs with a pGL3 vector in which the open-reading frame of luciferase reporter gene was placed downstream of the miR-302 promoter (pmiR-302)-Luciferase sequence. Un-transfected cells and cells transfected with a promoter less-Luciferase PGL3 vector served as negative controls. Measuring the emitted luciferase signal with a luminometer demonstrated a significantly elevated signal in the cell lines transfected with pmiR-302-Luc compared with those transfected with promoter less-Luc vector. It should be noted that while BMSCs were used as a negative control, it showed a low but significant activity of miR-302 promoter. Also note that despite an apparent elevation in the intensity of signal for the 1321N1 cell line, the difference was not statistically significant, due to the high variance in different experiments. BMSCs; Bone marrow stromal cells, *; P<0.05, **; P<0.01, and ***; P<0.001.

To quantitate the data, we used another construct in which the miR-302 promoter was placed upstream of the open-reading frame of the luciferase gene. As shown in figure 3, luciferase assay detected a significantly elevated signal in cell lines transfected with miR-302 promoter-Luc vector compared with those transfected with the control (promoterless-Luc vector) (p<0.05). Interestingly, while we used BMSCs as a negative control, the cells showed a much lower but still significant promoter activity (p<0.05).

### Serum deprivation induced formation of tumorospheres in glioblastoma cell lines

**Fig 4:**
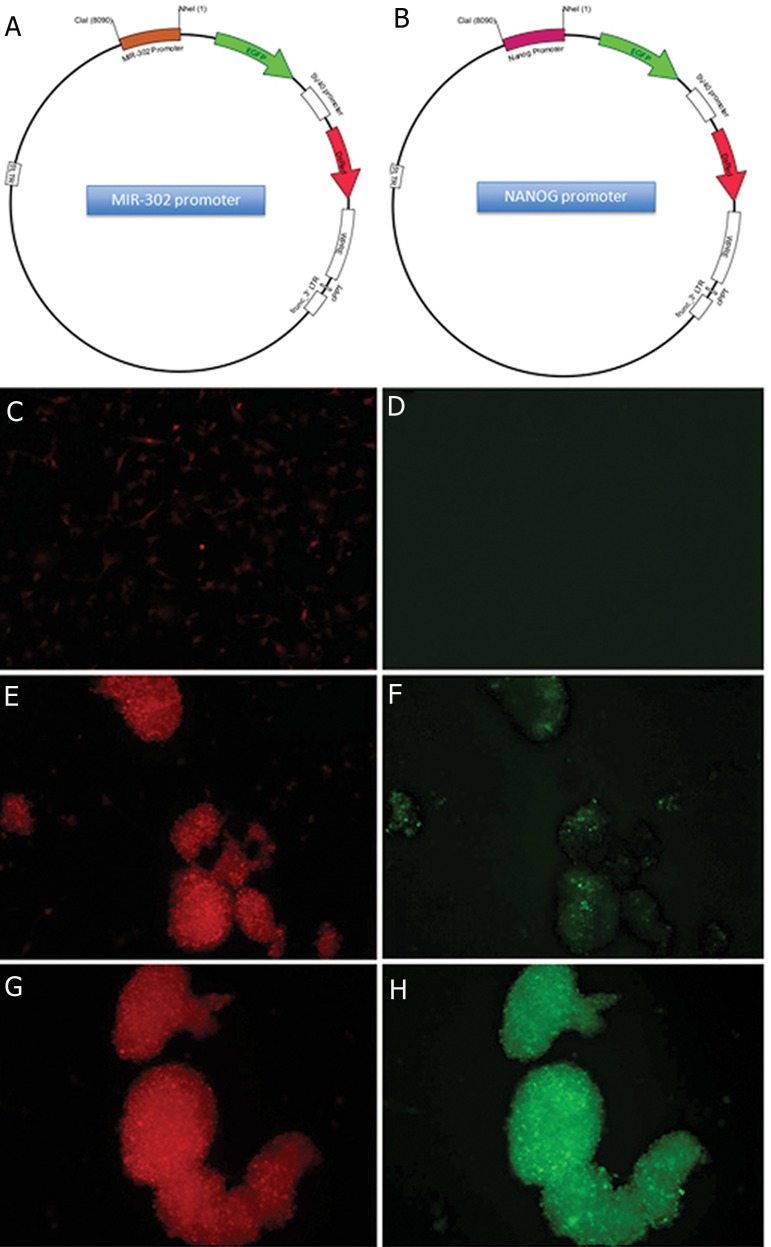
Activation of miR-302-367 and Nanog promoters after serum starvation of U87MG. MiR-302-367 promoter (A) and Nanog promoter (B) were cloned upstream of EGFP in a lentiviral vector, containing SV40-mCherry as a control of transduction. Almost all U87MG cells were transduced with the vector (C), however, no EGFP positive cell was detected (D). Following starvation and sphere formation, many EGFP+ cells were observed as a result of reactivation of the miR-302-367 (E, F) and Nanog (G, H) promoters. EGFP; Enhanced green fluorescent protein.

Using the luciferase assay, it was possible to look at the expression level of miR-302s in glioblastoma and medulloblastoma cell lines under different cell culture conditions. Interestingly, while U87MG cells infected with miR302-367 promoter-GFP/SV40 promoter-DsRed lentiviral vectors (Fig 4A, B) showed barely detectable GFP-positive cells (Fig 4C, D), treating the cells with serum-free media containing the G418 antibiotic generated GFP-positive colonies (Fig 4E, F). Repeating the experiment with Nanog promoter-GFP/SV40 promoter-DsRed lentiviral vectors proved that the same generated colonies have the property of stem-like cells (Fig 4G, H). Similarly, the data revealed that the promoter activity of miR-302s was significantly elevated in cells cultivated in serum-free medium. Cultivating the glioma cell lines under serum-free conditions for a couple of weeks led to the preferential survival of a subpopulation of cells, which eventually generated large and floating colonies (Figes4E-H, 5A, B).

**Fig 5:**
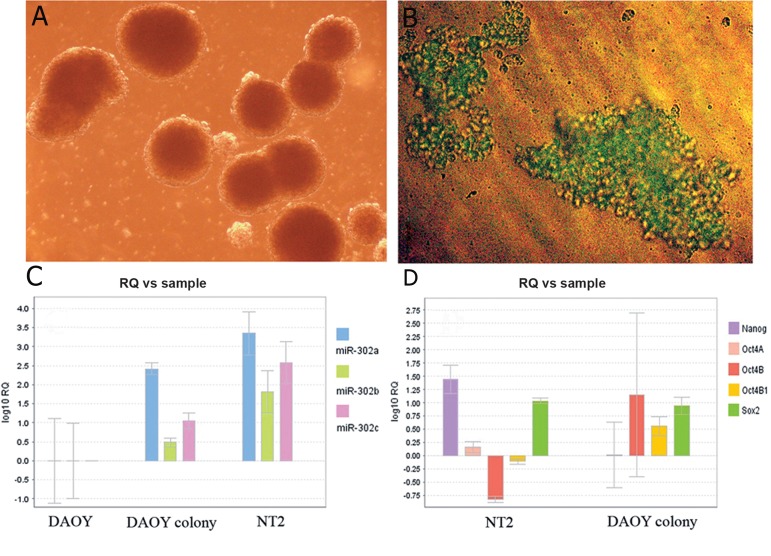
Formation of big and floating tumorospheres in serum-deprived DAOY cell line (A). The colonies could survive for several weeks in serum-free media. In panel B, the cells had been transfected with the pmiR-302-GFP plasmid prior to the serum removal and addition of G418. Note that most of the cells within the colonies are GFP-positive. C. Comparative miR-302s expression in DAOY cell line before and after colony formation. Histograms show a significant upregulation of members of miR-302 cluster (miR-302a, miR-302b, and miR-302c) in tumorospheres obtained from serum-deprived DAOY cell line. Note that the pattern of miR-302s upregulation is similar to that of the NT2 cell line. D. Upregulation of some ES-specific transcription factors in serum-deprived DAOY cells. Note that OCT4B1, Sox2 and to a lesser extent OCT4A, but not Nanog, are upregulated in tumorospheres obtained from serum-deprived DAOY compared with the untreated cells. The expression level of the genes was also compared to that of pluripotent NT2 cell line. GFP; Green fluorescent protein and ES; Embryonic stem.

The colony formation was not restricted to the serum deprived glioma cell lines as the same phenomenon was also observed in the medulloblastoma cell line DAOY (Fig 5A, B). To further determine whether the DAOY colonies are indeed an enriched population of miR-302 positive cells, total RNA was extracted from untreated DAOY cells as well as serum-deprived DAOY colonies and the expression level of miR-302s and some known ESC-specific transcription factors were compared in these samples. As demonstrated in figure 5C, miR-302A, miR-302B and miR-302c were significantly upregulated in serum-deprived DAOY colonies (p<0.001). Among the expressed miRs, miR-302a showed the highest upregulation whereas miR-302b displayed the lowest level. Interestingly, these patterns of expression were almost identical to those obtained for the NT2 cell line (Fig 5C).

Furthermore, the expression levels were also compared for the main transcription factors involved in self-renewal and pluripotency of stem cells, namely OCT4, Sox2 and Nanog. Except for Nanog, the expression of other factors was upregulated in DAOY colonies (Fig 5D). Among OCT4 variants, OCT4B and OCT4A showed the highest and lowest level of upregulation respectively. The expression level of OCT4B and OCT4B1 variants in NT2 cells was even lower than that in untreated DAOY cells.

## Discussion

There is accumulating evidence on the reexpression of embryonic genes in tumor cells (1, 2). Identifying such re-expressed genes in cancers could shed more light on the biology of tumor cells and in turn could help us to find more suitable tumor markers for better diagnosis and more efficient treatment of cancers. A good example of such genes is OCT4, a master regulator of self-renewal and pluripotency, which is exclusively expressed in embryonic stem cells (ESC) (18-20). Motivated by the cancer stem cell (CSC) concept, we have recently demonstrated a re-expression of OCT4, in bladder (21) and gastric (22) cancers. The literature on expression of OCT4 in cancer cell lines and tissues (23, 24) appears to be highly inconclusive due to the presence of several expressed OCT4 pseudogenes (25, 26) and the failure of techniques to discriminate between the expressions of different variants of OCT4 (14). Therefore, finding a better ESC-specific marker may result in a more valid and reproducible mean to evaluate the pluripotency state of stem and cancer stem cells in labs and clinics.

In the present study, we examined the potential expression and function of miR-302s, an ES-specific microRNA cluster, in four different brain tumor cell lines. The cluster of miR-302 is the most abundantly expressed set of miRNA in undifferentiated ESCs and its expression is sharply turned off upon the induction of differentiation (27). Indeed, the promoter of miR-302-367 cluster has binding sites for the main ESC-specific transcription factors, i.e. OCT4, Nanog, Sox2 and Rex1 (27, 28). The members of the cluster regulate cell cycle in ESCs and promote self-renewal and pluripotency of the cells and hence participate in the maintenance of ESCs (28, 29). However, their potential role in inducing pluripotency pathways in somatic cells for generation of cancer stem cells and initiation of tumorigenesity is still ambiguous.

Our data showed a very low expression of members of the miR-302s cluster in glioma cell lines in comparison with the embryonic carcinoma cell line NT2. Our data is in agreement with a similar finding by Lavon et al. (30) who compared the microRNA expression profile of a pool of glioma samples with those of ESC and neural precursor cells (NPCs). They found a much lower expression of miR- 302s in glioma cells compared with ESCs, but similar to that of NPCs. Moreover, our data revealed that the expression of miR-302 is restricted to a rare subpopulation of the cells. Further examinations revealed that the given subpopulation has the stemness property and presumably contains cancer stem cells. While a link between miR-302 and stemness state has already been reported (27-29, 31), to best of our knowledge, this is the first report to identify miR-302 as a potential cancer stem cell marker. Nevertheless, further work is needed to isolate and characterize the stemness property of the serum-deprived miR-302 expressing glioma cells before confirming their CSC nature.

Initially, we used BMSCs (an adult stem cell) as a negative control, but we discovered a low, but significant, miR-302 expression in this cell line. Based on this observation, it seems that there is a subpopulation of pluripotent cells within these heterogeneous cell populations. This is in accordance with previous reports, suggesting the existence of such a subpopulation within BMSCs (32). Interestingly, one simple way for enriching this subpopulation is via serum deprivation (32).

The miR-302 promoter-Luc assay generated a more quantitative data, compared with the miR-302 promoter-GFP assay, in deciphering the functional activity of miR-302 promoter in the cell lines employed. Furthermore, it confirmed the data produced by real-time PCR. This confirmation was necessary to rule out non-specific amplification of related sequences. While the promoter of miR-302-367 cluster is exclusively functional in pluripotent cells, the ES-specific expression of other members of this family (miR-302e and miR-302f; located on a different chromosome and with a difference of only one nucleotide from miR- 302 cluster members) has not been elucidated.

Using microRNA as a CSC, or more generally as a tumor marker, has several advantages compared with mRNA or protein markers. Firstly, due to their small sizes, they are very stable molecules under harsh conditions. This property of microRNAs could make them ideal markers in different clinical samples including serum (33), urine (34) and formalin-fixed Paraffin- embedded (FFPE) specimens (35). Secondly, due to their small size, they are unlikely to induce an immune response when administered to patients. This also makes microRNAs or their complementary strand an ideal mean for gene therapy (36).

For detection of miR-302s, we used locked nucleic acid (LNA) primers, which provided high level of specificity for the detection of miRNAs. As expected for a positive control, we detected a high expression level of miR- 302s in the NT2 cell line. While all members of the miR-302 cluster were transcribed under a common promoter, the level of expression varied among them. This could be due to the variation in the efficiency of different primers to specifically amplify individual members or due to the innate differences in the stability of mature members after being generated (37).

## Conclusion

Altogether, we used serum deprivation to enrich putative CSCs from several brain tumors, as described previously by others (38). Our data identify the members of the miR-302 cluster as potential CSC markers with potential diagnostic and therapeutic applications in glioma and probably other cancer types.
